# Xiaokeping Mixture Attenuates Diabetic Kidney Disease by Modulating TGF-*β*/Smad Pathway in db/db Mice

**DOI:** 10.1155/2019/9241896

**Published:** 2019-10-08

**Authors:** Bo Yang, Zhongni Xia, Chuanwei Xin, Chenggang Ma, Feng Zhang

**Affiliations:** ^1^Department of Pharmacy, Zhejiang Academy of Traditional Chinese Medicine, Tongde Hospital of Zhejiang Province, Hangzhou 310013, China; ^2^Department of Medical Engineering, Zhejiang Academy of Traditional Chinese Medicine, Tongde Hospital of Zhejiang Province, Hangzhou 310013, China

## Abstract

Xiaokeping mixture (XKP), a traditional Chinese medicine compound preparation, has achieved widespread use for diabetes mellitus and its kidney damage in clinical practice. The current study was carried out to assess the protective effect of XKP in spontaneous diabetic db/db mice and the underlying mechanism whereby XKP regulates TGF-*β*/Smad pathway. Male C57BLKS/J db/db mice, 12 weeks old, were randomly divided into 3 groups: the model group, 17.5 mg/kg irbesartan-treated group (IST group), and 8 g/kg XKP-treated group (XKP group), while age-matched db/m mice were selected as a control group. After 8 weeks of administration, serum and urea samples were collected from mice for biochemical tests, while the kidneys were removed for histological analysis. The expression of TGF-*β*/Smad pathway-related mRNA and protein were measured by RT-PCR and western blot analysis. Treatment with XKP significantly improved renal function and attenuated the pathological change of diabetic kidney disease (DKD) in renal histopathology. Furthermore, the overexpression of TGF-*β*1, Smad3, and p-Smad3 was inhibited, as well as the reduction of Smad7 and SIP1 was weakened by XKP. In conclusion, these results suggest that XKP could attenuate DKD by modulating TGF-*β*/Smad pathway.

## 1. Introduction

Diabetic kidney disease (DKD) is one of the most prevalent and severe chronic microvascular complications of diabetes mellitus that eventually leads to end-stage renal failure [1]. A major pathological change in DKD is characterized by abnormal metabolism of extracellular matrix components (ECM) [2]. As a critical cell regulatory factor, TGF-*β* plays a key role in the synthesis and accumulation of ECM in mesangial cells [3]. When various damage factors (such as high glucose levels) appear, TGF-*β*1 appears to be overexpressed, induces changes in mesangial cell function and morphology, and promotes increased secretion of ECM [4]. Further, blocking the activity of TGF-*β*1 contributes to attenuate DKD.

TGF-*β*1 binds to two transmembrane receptors, type I (TGF-*β*R1) and type II (TGF-*β*RII), forming a heterotetramer that leads to phosphorylation of Smad2 and Smad3. Then associated with Smad4, heteroligomerize is formed and translocates into the nucleus where it is involved in the transcription of TGF-*β*-responsive genes [5, 6]. Additionally, Smad7 is a negative regulator that could rapidly regulate the feedback mechanism of TGF-*β* response by competitively binding to TGF-*β*R1 and disrupting heteroligomerize [7].

Xiaokeping mixture (XKP), comprising Radix Astragali (*Astragalus membranaceus* (Fisch.) Bge), Rhizoma Dioscoreae (*Dioscorea oppositifolia* L.), Radix Rehmanniae Preparata (*Rehmannia glutinosa* (Gaert.) Libosch. ex Fisch. et Mey.), Radix Ophiopogonis (*Ophiopogon japonicus* (Linn. f.) Ker-Gawl.), Radix trichosanthis (*Trichosanthes kirilowii* Maxim.), Radix Salvia MiltiorrHizae (*Salvia miltiorrhiza* Bunge), Flos Chrysanthemi (*Dendranthema morifolium* (Ramat.) Tzvel.), and Fructus LyCii (*Lycium barbarum* L.), has achieved widespread use for diabetes mellitus and its kidney damage in clinical practice for decades [8,9]. Recent studies have demonstrated that XKP can effectively attenuate DKD in a rat model by inhibiting the expression of VEGF and ETS-1 [10]. XKP showed pancreatic *β*-cells protection against apoptosis under high glucose stress through mTOR-mediated autophagy pathway [11]. By restraining the activation of miR-192, XKP could be used to treat DKD induced by STZ [12]. Astragaloside A, one of the main active ingredients of XKP, has an antioxidation and hepatoprotection effect through downregulating the TGF-*β*/Smad signaling pathway [13]. Some herbs in XKP, such as Radix Astragali, Salvia MiltiorrHizae, and Fructus LyCii, can modulate the expression of TGF-*β* in animal experiment [14–16].

Hence, it is essential to understand the underlying mechanisms of XKP in the treatment of DKD. In this study, we employed spontaneous diabetic db/db mice to investigate the effects of XKP on the TGF-*β*/Smad pathway.

## 2. Materials and Methods

### 2.1. Reagents

Xiaokeping mixture was obtained from Tongde Hospital of Zhejiang Province (Hangzhou, China).

### 2.2. Experimental Animals and Treatment

All experiments were performed in accordance with the guidelines on ethical standards for investigations in animals, and the study was approved by the Tongde Hospital of Zhejiang Province animal research committee (No. XMSC2017024). 11-week-old male C57BLKS/J db/db and their age-matched db/m mice weighing 42.7 ± 2.8 g were purchased from Shanghai Slaccas Inc. (Shanghai, China). All mice were housed in a climate-controlled room at 24°C and 50% humidity under a 12 h light/dark cycle and had free access to water in the Animal Center of Zhejiang Chinese Medical University. At 12 weeks of age, the db/db mice were randomly divided into three groups (*n*=12 for each group) and orally administered, respectively, with saline (model group), 17.5 mg/kg irbesartan (IST group), and 8 g/kg XKP (XKP group). 12 male db/m mice, as control, were treated with an equivalent volume of saline. At 20 weeks of age, blood samples were drawn from aortaventralis, and urine samples were collected for biochemical detection. Mice were sacrificed by cervical dislocation, and kidneys were removed for histological, polymerase chain reaction (PCR) and western blotting assays.

### 2.3. Blood Biochemical Analysis

Blood glucose level, blood urea nitrogen, and serum creatinine were measured by an Automated Biochemical Analyzer (Hitachi, Japan).

### 2.4. Urine Analysis

In the end of experiment, mice were transferred to the metabolic cages for 24 h where urine samples were collected. Urine creatinine and albumin concentrations were determined by an Automated Biochemical Analyzer (Hitachi, Japan).

### 2.5. Renal Histological Analysis

Kidney sections were fixed in 4% buffered paraformaldehyde, embedded in paraffin, and cut into 4 *μ*m thick sections which were prepared for HE staining. Ten glomeruli for each mice were evaluated in a blinded analysis in kidney sections. The degree of glomerulus damage was assessed by a semiquantitative method as follows: no damage (grade 0, normal glomeruli); minimal damage (grade 1, mesangial expansion area up to 25%); moderate damage (grade 2, 26–50% expansion); moderate-to-severe damage (grade 3, 51–75% expansion); and severe damage (grade 4, 76–100% expansion). The glomerular matrix index (GMI) was then calculated by the following formula:

(1) $$\begin{array}{c} \text{GMI}=\frac{1\times n_{1}+2\times n_{2}+3\times n_{3}+4\times n_{4}}{n_{0}+n_{1}+n_{2}+n_{3}+n_{4}}. \end{array}$$

### 2.6. Analysis of TGF-*β*1, Smad3, Smad7, and SIP1 mRNA Expressions by Reverse Transcriptase-PCR

Total RNA was extracted from kidney tissues using the Trizol reagent (Sangon Biotech, Shanghai, China) as described in the instruction. Reverse transcribed to cDNA was conducted following the instruction of reverse transcription kit (ComWin Biotech, Beijing, China). The primer sequences, designed and synthesized by ComWin Biotech Co., Ltd (Beijing, China), are listed in Table 1. The conditions of PCR cycling were as follows: predenaturation at 95°C for 10 min, 40 cycles at 95°C for 15 s, and 60°C for s60. The relative expression level of target gene was determined by 2^−ΔΔCt^ method. Following RT-PCR, melting curve analysis was performed immediately for homogenization.

### 2.7. Western Blot Analysis for TGF-*β*1, Smad3/7, p-Smad3, and SIP1

Renal tissues were homogenized in radioimmunoprecipitation assay (RIPA) lysis buffer (200 mg tumor tissue/1 ml RIPA, Beyotime Biotechnology, Shanghai, China) for 30 min on ice, and then centrifuged at 12,000 rpm for 5 min, supernatants were collected. The protein concentration was determined by the bicinchoninic acid (BCA) protein assay (Solarbio, Beijing, China). Protein samples were separated by electrophoresis on 5% SDS-PAGE gels and transferred to polyvinylidene difluoride (PVDF) membranes. Blocked in 5% (W/V) nonfat milk for 1.5 h, the membranes were incubated with the primary antibody against TGF-*β*1 (Abcam, Cambridge, UK), Smad3 (Affinity Biosciences, Ohio, USA), Smad7 (Affinity Biosciences, Ohio, USA), SIP1 (Abcam, Cambridge, UK), p-Smad3 (Cell Signaling Technology, Massachusetts, USA), and GAPDH (Proteintech, Illinois, USA) at 4°C overnight. After washing three times in TBST, the membrane was treated with horseradish peroxidase (HRP)-linked secondary antibody (Cell Signaling Technology, Massachusetts, USA) for 1 h and then washed three times in TBST. Density of the corresponding bands was measured by using chemiluminescence detection reagents (Solarbio, Beijing, China) and analyzed with Quantity One software.

### 2.8. Statistical Analysis

Data analysis was performed using SPSS 19.0, and the data are expressed as means ± SD. The LSD method in one-way ANOVA was used to compare the two groups in different groups, and *P* < 0.05 was considered as significant.

## 3. Result

### 3.1. Effects of Xiaokeping Mixture on Biochemical Indicators

**Figure 1:**
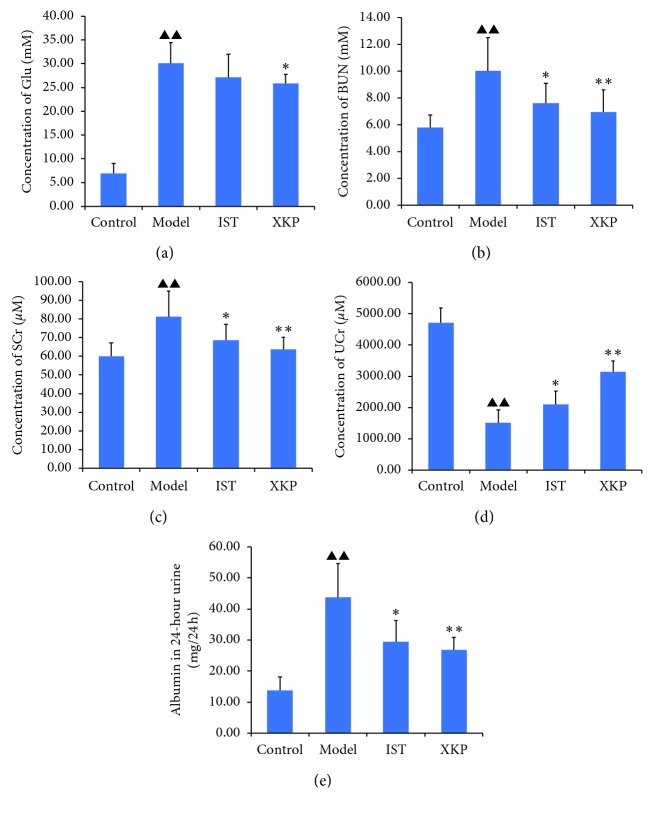
Effects of xiaokeping mixture on biochemical indicators. Mice in the model exhibited significantly increased level of blood glucose, and XKP has some effect on the regulation of blood glucose levels (a). XKP can prevent the elevation of blood urea nitrogen while it increases in the model group (b). Serum creatinine (c) and urine creatinine (d) both also increase in the model group, which reduced significantly by treatment of XKP. The same is true for the effect of XKP on urine albumin (e). Data presented are means ± SD. Compared with control, ^▲^*p* < 0.05, ^▲▲^*p* < 0.01. Compared with model, ^∗^*p* < 0.05, ^∗∗^*p* < 0.01.

There is no accidental death of mice occurred throughout the experiment. After 8 weeks of administration, there is a significant (*P* < 0.05) difference in blood glucose levels, blood urea nitrogen, serum creatinine, urine creatinine, and urine albumin between the control group and the model group (Figure 1). Furthermore, treatment with XKP significantly reduced the blood glucose levels (*P* < 0.05), blood urea nitrogen (*P* < 0.01), serum creatinine (*P* < 0.01), and urine albumin (*P* < 0.01) in db/db mice, while urine creatinine (*P* < 0.01) was significantly increased (Figure 1).

### 3.2. Effects of Xiaokeping Mixture on Renal Pathology

**Figure 2:**
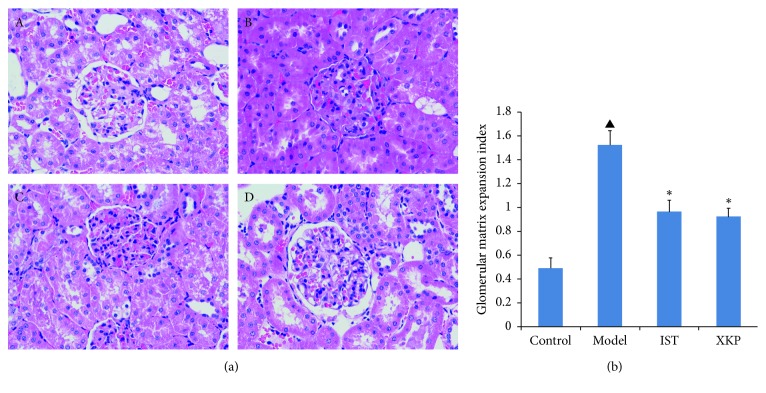
Effects of xiaokeping mixture on renal pathology. The pathological manifestations of typical DKD are mesangial cell growth without immune complex deposition; thickening of the dense layer of glomerular basement membrane is several times thicker than normal, diffuse increase of mesangial matrix, glomerular tuberous sclerosis, and renal arterioles Glassy change. (a) Representative photomicrographs of renal cortical sections from control group A, model group B, IST group C, and XKP group D. (b) Glomerular matrix expansion index scores (GMI) calculated from the analysis of 20 glomeruli per mouse. ^▲^*p* < 0.05, compared with control; ^∗^*p* < 0.05, compared with model (*n*=12 per group). HE staining, 400x.

As shown in Figure 2(A), the kidney tissue of the control group was intact and clear, the basement membrane was smooth, and no obvious pathological changes were found in the glomeruli. By contrast, many necrosis symptoms of DKD-induced kidney tissue have been observed in the model group such as basement membrane thickening, increased mesangial matrix, and mesangial cell proliferation (Figure 2(B)). XKP treatment attenuated the above symptoms (Figure 2(D)). The GMI score was significantly lower in the XKP group than in the model group.

### 3.3. Effects of Xiaokeping Mixture on the Expression of TGF-*β*1, Smad3, Smad7, and SIP1 at the mRNA Level

**Figure 3:**
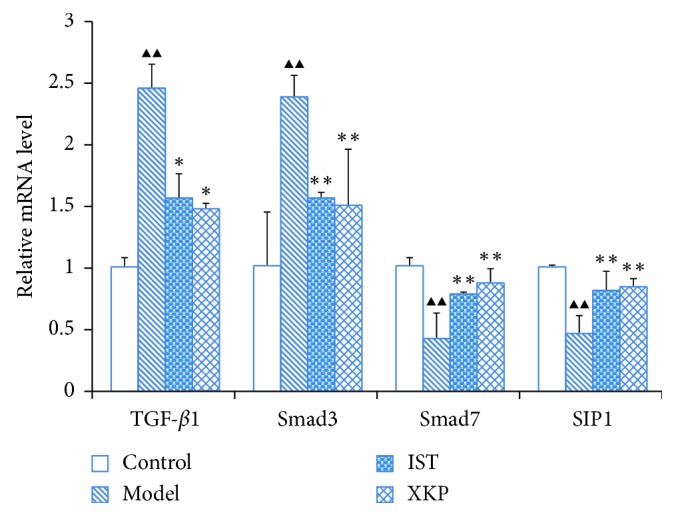
Effects of xiaokeping mixture on the mRNA expression of TGF-*β*1, Smad3, Smad7, and SIP1. XKP markedly modulated the mRNA expression of TGF-*β*1, Smad3, Smad7, and SIP1 in db/db mice, suggesting that XKP confers beneficial effect on attenuating DKD. Compared with control, ^▲^*p* < 0.05, ^▲▲^*p* < 0.01. Compared with model, ^∗^*p* < 0.05, ^∗∗^*p* < 0.01.

In order to evaluate the underlying mechanism of XKP in improving DKD, we measured the expression of TGF-*β*/Smad pathway-related mRNA by quantitative real-time PCR. The mRNA expression of TGF-*β*1 (*P* < 0.05) and Smad3 (*P* < 0.01) were significantly increased while the expression of Smad7 (*P* < 0.01) and SIP1 (*P* < 0.01) were significantly decreased in the model group compared to relative levels in the control group (Figure 3). However, treatment with XKP markedly attenuated these trends (Figure 3).

### 3.4. Effects of Xiaokeping Mixture on the Expression of TGF-*β*1, Smad3/7, p-Smad3, and SIP1 at the Protein Level

**Figure 4:**
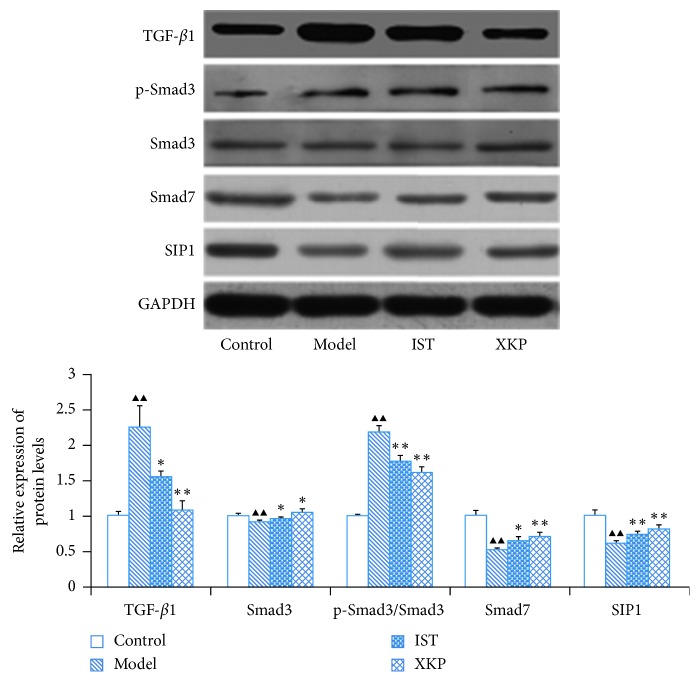
Effects of xiaokeping mixture on the protein expression of TGF-*β*1, Smad3/7, p-Smad3, and SIP1. Beneficial changes were observed in western blot analysis. XKP prevents deterioration of renal by regulating the expression of TGF-*β*/Smad pathway-related protein. Compared with control, ^▲^*p* < 0.05, ^▲▲^*p* < 0.01. Compared with model, ^∗^*p* < 0.05, ^∗∗^*p* < 0.01.

Western blot analysis was also used to verify the impact of XKP on DKD. Mice in the model exhibited significantly increased protein expression of TGF-*β*1 (*P* < 0.01) and Smad3 (*P* < 0.01), as well as markedly decreased protein expression of Smad7 (*P* < 0.01) and SIP1 (*P* < 0.01) compared with the control group. Just as the effects on mRNA expression, XKP also dramatically attenuates these deteriorating trends (Figure 4). Phosphorylation activation of Smad3 is one of the important markers of Smad signaling pathway activation. Thus, a balance between p-Smad3 and Smad3 reflects the progression of DKD. The expression of p-Smad3 was also examined, and its ratio to Smad3 showed a significant increase (*P* < 0.01) in the model group when compared with the control group. Similarly, XKP significantly (*P* < 0.01) inhibits phosphorylation of Smad3 (Figure 4).

## 4. Discussion

Diabetes kidney disease, one of the major microvascular complications of diabetes, is closely related to microvascular inflammatory response caused by long-term hyperglycemia [17]. This study suggests that treatment with XKP regulates the expression of TGF-*β*/Smad pathway-related mRNA and protein, thereby delaying DKD progression.

The db/db mouse, a spontaneous model of type 2 diabetes, carries a G-to-T point mutation of the leptin receptor on chromosome 4 that leads to hyperphagia, decreased energy expenditure, hyperglycemia, and insulin resistance [18]. Compared with the traditional STZ-induced DKD model, the db/db mouse model of type 2 diabetes is more stable and can reduce individual differences, which is considered to be an ideal animal model of diabetes [19, 20]. In general, the db/db mice develop abnormal glucose tolerance at 3-4 weeks of age, proteinuria at 7-8 weeks of age, obvious symptoms of diabetic nephropathy at 12 weeks of age, and renal histopathological features resembling those of human diabetic nephropathy [21]. Moreover, mesangial matrix diffuse increase, and glomerular tuberous sclerosis had been found in the HE staining of model mice, which was consistent with previous reports [22].

Many Chinese herbal medicines are considered to have anti-inflammatory effects and widely used in the treatment of chronic inflammatory diseases such as diabetes. According to traditional Chinese medicine theory, Radix Astragali, Rhizoma Dioscoreae, Radix Rehmanniae Preparata, Radix Ophiopogonis, Radix trichosanthis, Radix Salvia MiltiorrHizae, Flos Chrysanthemi, and Fructus LyCii are made into compound preparation for the treatment of diabetic kidney disease. Referring to the name of diabetes in traditional Chinese medicine theory, the compound preparation is named as xiaokeping mixture. The major constituents of xiaokeping mixture have been shown to regulate blood sugar levels and prevent complications of diabetes in animal experiments and clinical observation. Since Radix Astragali and Rhizoma Dioscoreae are one of the main components of XKP, they have a significant effect on lowering blood sugar [23]. As a result of lowering blood sugar, the glucose toxicity of db/db mice is alleviated, and diabetic kidney disease is relieved to some extent. In addition, some herbal medicine with the function of promoting blood circulation and removing blood stasis and collaterals is the basic treatment for diabetic kidney disease based on the theory of traditional Chinese medicine. And the two components of XKP, Radix Salvia MiltiorrHizae and Radix Rehmanniae Preparata have been confirmed to have the function of promoting blood circulation and removing blood stasis and collaterals in previous studies [24]. However, the renoprotective mechanism of XKP for diabetic kidney disease has not yet been fully defined. In the current study, XKP was demonstrated to have hypoglycemic effects while attenuating the progression of DKD. Blood urea nitrogen, serum creatinine, urine creatinine, and albumin in 24-hour urine are considered indicators for evaluating renal function. Renal deterioration usually manifests as elevated blood urea nitrogen, elevated serum creatinine, decreased urine creatinine, and albuminuria, which were observed in db/db mice. However, treatment with XKP reversed the above symptoms and improved the renal function. Pathological evidence further supports this result.

TGF-*β* is a cytokine that regulates cell growth and differentiation, including three isoforms *β*1, *β*2, and *β*3 [25]. Furthermore, the distribution of the TGF family in the kidney is dominated by TGF-*β*1, which regulates multiple pathways to influence the progression of renal fibrosis. As an important intracellular kinase substrate, Smad family proteins play a key role in the transmission of TGF-*β* signaling from cell surface receptors to the nucleus [3]. Therefore, we aimed to investigate the mechanism of XKP attenuating DKD by focusing on the TGF-*β*/Smad pathway. In the TGF-*β*/Smad pathway, Smad2 and Smad3 were known to accelerate renal fibrosis, whereas Smad 7 can inhibit TGF-*β* signal transduction to prevent fibrosis. In the current study, XKP can reduce the expression of TGF-*β*1 and Smad3 in the db/db mice and upregulate the expression of Smad7. Additionally, the regulation of TGF-*β*1 was inseparable from the activation and phosphorylation of Smad3. XKP also reduced the expression of p-Smad3, which provides further evidence for the effectiveness of XKP in the treatment of DKD.

SIP1, an abbreviation for Smad-interacting protein 1, was thought to play a role in the TGF-*β* signaling pathway. SIP1 could bind tightly to Smad 1, 2, 3, 5, and 8 via the Smad-interacting domain for SMAD interaction that acts as a cotranscriptional repressor [26]. SIP1 is also a member of the *δ*-EF1 family of two-handed zinc-finger factors (Zeb2), which have been described to directly repress transcription of target genes by binding to the E2 boxes (CACCTG sequence) in their promoter [27]. In the process of renal fibrosis, ECM and epithelial-to-mesenchymal transition (EMT) are very important pathological changes. These changes involve many cytokines such as E-cadherin, vascular endothelial growth factor (VEGF), connective tissue growth factor (CTGF), plasminogen activator inhibitor 1 (PAI-1), and matrix metalloproteinases (MMPs) [28, 29]. Interestingly, cytokines mentioned above are regulated by SIP1 [27, 30–33]. Obviously, these regulations are complicated and networked. Differential fine tuning of SIP1 in interstitial fibrosis may result in controversial functional consequences [34, 35]. In the current study, when compared with the control group, a marked downregulation of SIP1 expression in the kidney tissue of db/db mice was observed, which was consistent with previous studies [36]. Notably, treatment with XKP promoted the expression of SIP1, which was associated with improved renal function.

## 5. Conclusions

In conclusion, we demonstrated the protective role of XKP in type 2 diabetic db/db mice. XKP could effectively regulate blood sugar levels and attenuate DKD, which is likely related to inhibition of TGF-*β*/Smad pathway.

## Figures and Tables

**Table 1:** PCR sequences and PCR products.

Name	Upstream primer (5′–3′)	Downstream primer (5′–3′)
TGF-*β*1	ATTCCTGGCGTTACCTTGG	AGCCCTGTATTCCGTCTCCT
Smad3	GCCCAGTTACCTACTCGG AGC	TGTTGACATTGGAGAGCAGC
Smad7	TACCGTGCAGATCAGCTTTG	TTTGCATGAAAAGCA AGCAC
SIP1	TGAAGATGAAGAAGGCTGGAA	GCAAGGGAGGAAAACCAACT
GAPDH	ATGACTCTACCCACGGCAAG	TACTCAGCACCAGCATCACC

## Data Availability

The data used to support the findings of this study are available from the corresponding author upon request.
